# Lignin metabolism involves *Botrytis cinerea* BcGs1- induced defense response in tomato

**DOI:** 10.1186/s12870-018-1319-0

**Published:** 2018-06-04

**Authors:** Chenyu Yang, Yingbo Liang, Dewen Qiu, Hongmei Zeng, Jingjing Yuan, Xiufen Yang

**Affiliations:** The State Key Laboratory for Biology of Plant Disease and Insect Pests/ Key Laboratory of Control of Biological Hazard Factors (Plant Origin) for Agri-product Quality and Safety, Ministry of Agriculture Institute of Plant protection, Chinese Academy of Agricultural science, No. 12 Zhong-guan-cun South Street, Beijing, 100081 China

**Keywords:** Fungal protein elicitor, Defense response, iTRAQ, Phenylpropanoid, Lignin, *Botrytis cinerea*

## Abstract

**Background:**

BcGs1, a cell wall-degrading enzyme (CWDE), was originally derived from *Botrytis cinerea*. Our previous study revealed that BcGs1 could trigger defense responses and protect plants against various pathogens. We researched the defense response mechanism underlying this BcGs1 elicitation in tomato.

**Results:**

We revealed that the two domains were required for BcGs1’s full necrosis activity. According to analysis and quantitative real-time PCR of the up-regulated proteins and genes filtered by iTRAQ-based quantitative proteome approach, oxidative metabolism and phenylpropanoid metabolism were speculated to be involved in BcGs1-triggered defense response in tomato. Furthermore, experimental evidence showed that BcGs1 triggered reactive oxygen species (ROS) burst and increased the level of phenylalanine-ammonia lyase (PAL) and peroxidase (POD) enzyme activity, as well as lignin accumulation. Moreover, histochemical analysis revealed that infiltration of BcGs1 in tomato leaves exhibited cell wall thickening compared with untreated plants.

**Conclusions:**

The results suggested that BcGs1 activated the basal defense response included lignin metabolism contributed to BcGs1-induced resistance to *Botrytis. cinerea* infection in tomato.

**Electronic supplementary material:**

The online version of this article (10.1186/s12870-018-1319-0) contains supplementary material, which is available to authorized users.

## Background

Plants intimately interact with various microbial pathogens in the complex nature environments. To protect themselves against microbial colonization, plants have evolved a variety of defense mechanisms, including constitutive and inducible resistance strategies. Most potential pathogens are prevented by preformed physical and chemical barriers or after the induction of a complex array of defense responses. In order to Start the relevant resistance system in plants a specific receptor to identify the pathogen is required, at the same time, the plant cell wall-derived molecules may be involved [1]. Following pathogen recognition, which is identified by pathogen-associated molecular patterns (PAMP), microbe-associated molecular pattern (MAMP)-triggered immunity (PTI) or effector-triggered immunity (ETI) through immune recognition systems [2–4], the protein kinase would be activated and ROS would be accumulated [5, 6]. As a chain reaction, downstream signal including the defense-related genes and PR proteins would be activated [7, 8]. Plants rely on the basal defense response by using a specific recognition system to prevent penetration and restrict the growth of pathogens, such as cell death, oxidative burst, defense genes, PR protein expression, phytoalexins, callose deposition, lignification and cell wall thickness [2].

To overcome the barrier of the plant cell wall, phytopathogenic fungi secrete various CWDEs, such as cellulases, pectinase, hemicellulases, cutinase and protease. Most of these enzymes not only degrade cell wall components to get carbon sources for pathogen growth but can also trigger multiple plant defense responses.

During infection, necrotrophic pathogens first kill host cells and/or feed on dead tissue. The necrotrophic pathogen *Botrytis cinerea* often secretes non-host-selective toxins, CWDEs and proteinases to facilitate host cell death [9]. Although plant cell death that resulted from biotrophic and necrotrophic pathogen infection plays a central role in multiple defense responses, it has markedly different roles in plant responses to necrotrophs and biotrophs that are dependent on plant-pathogen interaction. The cell death induced by necrotrophic pathogens is more complex than that of biotrophic pathogens in plant immunity because necrotrophic fungi have subtler pathogenic tactics [10]. Variation in multiple basal defense mechanisms is thought to underlie differences in host susceptibility to necrotrophic pathogens. Although the genome of fungus was well-known and *Botrytis cinerea* biology has been extensively studied [11, 12], the understanding about the biological processes of plants response to *Botrytis cinerea* is also very limited. Two elicitors isolated from *Botrytis cinerea*, namely botrycin and cinerein, caused the formation of necrotic lesions, rapid transcriptional activation of genes encoding enzymes of the phenylpropanoid pathway and distinct mitogen-activated protein kinases (MAPKs) in grapevines (*Vitis vinifera* L) [13]. However, the mechanisms of most secretory elicitors from *Botrytis cinerea* that trigger the plant defense response are unclear.

CWDEs stimulate multiple immune responses in plants and play a special role in pathogen-host plant interactions, but the mechanism is very complex. Some CWDEs triggering defense responses are associated with their degrading enzyme activity, but some are not related to enzyme activity [14]. *Botrytis cinerea*-produced polygalacturonases (PGs) and fungal xylanase (Xyn11A) can induce phytoalexin, ethylene and pathogenesis-related protein synthesis and show an immune resistance against *Botrytis cinerea* [14–17]. Present evidence suggests that these fungal CWDEs trigger typical PTI directly or indirectly through the perception of damage-associated molecular patterns (DAMPs) and through plant cell wall fragments generated by the CWDE degradation [10]. PTI activates a basal defense response, such as the biosynthesis of antimicrobial secondary metabolites (e.g., phytoalexins) and the expression of defense-related proteins, including pathogenesis-related proteins (PR), cell wall lignifications, protease inhibitor expression, and hormone biosynthesis [18, 19]. Ultimately, plants exhibited broad-spectrum resistance to fungi, bacteria and viruses [16].

*Botrytis cinerea,* which is one of the most destructive diseases worldwide, is a typical necrotrophic pathogen and causes gray mold disease in tomatoes, strawberries, grapes, cucumbers, soybeans and sunflowers [20]. Resistance breeding efforts have not met with success for botrytis diseases, although genetic resistance is effective and sustainable to protect plants. Elicitors could improve plant resistance to pathogens and are an alternative strategy to reduce plant disease [21]. Understanding the interaction of necrotrophic pathogens and host plants will provide an insight for elicitor application. However, plant basal defense response triggered by many single purified protein elicitors, including BcGs1, has not been clear. In this study, we first described the differential-display defense protein by the iTRAQ method. Based on global differential protein, phenylpropanoid metabolism was implied to be involved in BcGs1-induced tomato defense responses to *Botrytis cinerea*. Previous studies have shown Phenylpropanoid metabolism played an important role in cotton induced resistance to *V. dahlia* [22]. Meanwhile, the obvious differences including protein expression, lignin metabolism level and cell wall thickening, between BcGs1-induced plants and control were verified in late experiment. This study will help to understand the interaction between necrotrophic fungal pathogens and host plants and provide a theoretical basis for gray mold disease management through elicitor-activating plant immunity.

## Methods

### The fungal pathogen cultures and plant cultivates

*Botrytis cinerea* strain BC-98 was originally isolated from diseased tomato tissues at the Beijing Region, PR China. The fungus was maintained on potato dextrose agar (PDA) medium and cultured in Czapek-Dox liquid medium on a rotary shaker at 25 °C. Tomato seedlings (Zhong za 9, Purchased in Vegetable Flower Research Institute, Chinese Academy of Agricultural Sciences) were grown in a greenhouse at 24–28 °C with 70–80% relative humidity, and dark/light ratio was 10/14 h.

### Necrosis activity of BcGs1

Protein BcGs1 was obtained by the method described by Zhang et al. [23]. The necrosis-inducing activity of BcGs1 was observed at 12 h post injection of tomato, tobacco, cucumber and pea leaves with 1 μM BcGs1 by a 1 ml syringe, while Tris-HCl buffer (50 mM) was used as a control.

### Bioassay for BcGs1-induced disease resistance in tomato

Six to eight tomato plant leaves were injected with 250 nM BcGs1 protein solution (10 μL) and Tris-HCl buffer (50 mM). The same growing leaves were disinfected with alcohol and then rinsed with distilled water at 48, 72, 96, 120 and 168 h post BcGs1 injection, respectively. The two leaves were placed in a Petri dish with wet filter paper and petiole were moisturize with wet cotton wool. *Botrytis cinerea* disc was placed on the leaves and incubated for 48 h under continuous light and 100% humidity at 25 °C in a chamber, then lesion diameter was measured using vernier caliper. The induced disease resistance was calculated using the formula: Disease reduction (%) = [(size of lesions on control leaves-size of lesions on elicitor treated leaves)/size of lesions on control leaves] × 100 and the results were analyzed by statistical analysis software. Fifteen tomato plants were used in each treatment and control and three leaves were taken from per plant. Three times were repeated.

### Identification of functional domain of BcGs1

Biological information analysis showed that BcGs1 contained two domains of Glyco-hydro 15 (GH15) and CBM20_glucoamylase (CBM20). To identify its active structure, the transient expression vector pYBA1152, which contains a fluorescent protein and could fuse to my protein/domains of the protein, was used to express protein BcGs1, GH15 and CBM20 in *Nicotiana benthamiana* through an Agrobacterium-mediated approach. Fluorescence confocal microscopy and western blot analysis were performed to detect the expression at 48 h after injection of the recombinant *Agrobacterium*. At the same time, the necrosis activity was observed with the naked eye.

### Western blot

The sample leaves of *Nicotiana benthamiana* were collected after injected the *Agrobacterium* carrying recombinant gene of pYBA1152-BcGs1/GH15/CBM20 48 h. BcGs1, GH15 and CBM20 gene sequences were constructed to carry a His-tag, respectively. The tree proteins were extracted from the sample, separately, using plant total protein extraction reagent (Purchased from Biotechnology Company). The total proteins were subjected to SDS-PAGE in three lanes after concentration determination and boiling. The PEVD membrane was used to carry the protein glue after SDS-PAGE, the electrotransfer instrument was applied for 1 h to transfer the proteins to PEVD membrane. The PEVD membrane was incubated with blocking solution for another 2 h after washing with TBST buffer. Anti-His Tag Mouse Monoclonal Antibody was added and used to bind to His-tag and incubated for 2 h, after that HRP Conjugated Anti-His Tag Monoclonal Antibody was applied to bind to Anti-His Tag Mouse Monoclonal Antibody for 1 h. The BCIP/NBT was mixed well on PEVD membrane for color. At last, the membrane was put into the instrument to exposure and take pictures.

### Differential display protein analysis

Six to eight tomato plant leaves were injected with 1 μM BcGs1 protein solution (10 μL), and Tris-HCl buffer (50 mM) was used as a control. Tomato leaves were collected 24 h after treatment and used for proteomics detection by iTRAQ (Bangfei Biotechnology, Beijing, China). Each sample was repeated three times. A protein ratio > 1.3 or < 0.77 and *P*-value < 0.05 was regarded as being differentially expressed. Gene ontology (GO) analysis was applied to predict the protein function and calculate the functional category distribution frequency. KEGG analysis was conducted to analyze the functional protein annotation.

### Quantitative real-time PCR

The tomato leaves were injected with BcGs1 and Tris-HCl buffer. Total RNA was extracted at 0, 6, 12, 24, 48, 72, 96 h post treatment according to the protocol of the plant RNA Kit (Tiangen Biotech, Beijing, China). First-strand cDNA was synthesized from the total RNA using SuperMix for qPCR (TransGen Biotech, Beijing, China). The Applied Biosystems7500 Real Time PCR System was used to perform amplification with SYBR Green SuperMix. The primers (Table 1) were designed by Beacon Designer 8.1. Actin, an internal reference, was used to normalize the amount of cDNA in each reaction. All the qPCR was repeated three times to calculate the average values for quantification. Each reaction melt curve was analyzed, and a negative control without cDNA template was run with each reaction to evaluate the primer specificity. The relative gene expression levels were calculated from the average values by ΔΔCt method.

**Table 1:** Primers for quantitative validation of differentially expressed proteins induced by BcGs1

Names	Forward primers(5 - > 3)	Reverse primers(5 - > 3)
PR1	ATCATTTGTTTCCTTACCTTTG	ACTCCAACTTGTCTACGA
PR10	TTACAAGACAACAACTGAGTAT	AGCGTAGACAGAAGGATT
PR-Leaf 4	GACTATCTTGCGGTTCAC	GCTCTTGAGTTGGCATAG
NP24	TTGTTCTCTTCTTCCTTCTT	GGTGTATGGACAGTTGTT
PRSTH-2	TGTGTTGAAGGATGAAGAA	TAAGCGTAGACAGAAGGA
PRSTH-2-like	CTCCACCATCTCCTTGTA	ACACCAATTCGTTTATTTAAGG
Endo chitinase EP3	TGTTGGTTCTACTGATGAT	GGTAATCTGTGTTGTTCTC
Glucan endo-1,3-β-glucosidase B	ATGCTATGTTGGATTCTGTT	TTCTCGGACTACCTTCTTTA
Peroxidase1	ACTTCTCGTGCTAATAACAAT	CAGTAGTTGAGTCTCTTCTTC
Peroxidase 12	GGCTTACTTCGTCTTCATT	GACACAACTTGACCACAT
Peroxidase 21	TGTTATTACCTCTACTTCTTCAC	ATGTTGCCACTTGTTCTT
Peroxidase −2 like	GATGTTGTTCGGACCTATA	ATTACTATTCACCTTGCTACA
Peroxidase71	TGTCCTAATGTTGAATCCA	CTCCTGCCAATGATAGAT
ACC1	GTAATGGACACAGTAGAGA	GAGATATTAGAAGTAGGAAGATG
Auxin repressed/dormancy associated protein	GATGATGTTATGGCTGGT	GGTACTTGCTAGATCCTTC
Actin	GGTGTGATGGTGGGTATGG	GCTGACAATTCCGTGCTC

### H_2_O_2_ accumulation and formation in tomato leaves

H_2_O_2_ production was examined in 6–8 tomato leaves at 2, 4, and 6 h after treatment with 1 μM BcGs1 or Tris-HCl buffer. Leaves were excised and placed in water with 0.01% Triton-X-100 and 1 mg/mL nitro 3, 3′-diaminobenzidine (DAB); then, the solution was infiltrated with low vacuum pressure for 5 min, and the leaves were incubated overnight at room temperature. After that, the leaves cleared in alcoholic lactophenol (95% ethanol:lactic acid:phenol, 2:1:1) at room temperature until the leaves did not contain chlorophyll, and they were then rinsed with water. H_2_O_2_ can be visualized by a brownish-red precipitate that formed by polymerization with DAB.

H_2_O_2_ content was detected in the leaves using the H_2_O_2_ Test Kit (Jiancheng Biotechnology, Nanjing, China) at 0, 2, 4, 6, 12, and 24 h post-treatment. Samples of 150 mg were ground using a grinding machine and homogenized in extraction buffer (0.05 mM Phosphate buffer, pH 7.2). The supernatant was collected for H_2_O_2_ content detection after centrifugation at 10000 g for 10 min at 4 °C. The reagent provided by the kit was incubated with the initial solution following the manufacturer’s instructions. The H_2_O_2_ content was detected at 405 nm wavelength. Each experiment was repeated three times.

### Measurement of enzyme activities

The leaves treated with 1 μM BcGs1 and Tris-HCl buffer (as a control) were collected at 0, 12, 24, 48, 72, and 96 h, frozen in liquid nitrogen immediately and then stored at − 80 °C. One hundred to two hundred mg leaf samples were ground and homogenized in extraction buffer (1.0 mM Phosphate buffer, pH 7.4). The supernatant was collected for defense enzyme determination following the extraction kit. PAL activity was detected using a PAL Extraction Kit (Jiancheng Biotechnology, Nanjing, China) following the kit’s protocol. The supernatant applied to detect POD activity was collected at 3500 g for 10 min. POD activity was detected at 420 nm after mixture incubation.

### Lignin content detection in tomato leaves

The thioglycolic acid (TGA) method, a previously described method with modifications [24], was used to evaluate the lignin content in three biological replicates. Tomato leaves treated with 1 μM BcGs1 or Tris-HCl buffer were collected at 12, 24, 48, 72, 96, 120 and 144 h post BcGs1 treatment. Samples were homogenized and washed with 20 ml phosphate buffer (pH 7.8) and centrifuged at 5000 g for 10 min and the process was repeated three times. The pellet was dried at 80 °C for 24 h. One to two milligram of residue was weighed in tubes, mixed with 1.5 ml 2 N HCl and 0.3 ml TGA, and then incubated at 95 °C for 4 h after mixed well. The mixture was rapidly cooled on ice and centrifuged at 10,000 g for 10 min. The pellet was washed three times with 1 ml distilled water. Pellets were re-suspended with 1 ml 0.5 N NaOH and shaken at 200 rpm for 18 h at room temperature and centrifuged at 15,000 g for 10 min. The suspension was then transferred into a new tube, and the pellet was washed again with 0.5 ml 0.5 N NaOH. After centrifugation, the two supernatants were combined and mixed with 0.3 ml concentrated HCl. The mixture was incubated at 4 °C for 4 h to precipitate the lignothioglycolate derivates. After centrifugation, the pellet was solubilized in 1 ml 0.5 N NaOH. Absorbance of the resulting solution was measured at 280 nm.

### Change of cell wall morphology

Leaf samples from 6 to 8 leaf tomato plants were embedded using the previously described methods with some modifications [25]. Parts of the leaves around the BcGs1-induced necrotic spots were harvested at 3 d and 5 d post induction. Tris-HCl buffer was used as a control. The samples were then fixed in 1.0 ml phosphate buffer(pH 7.2) containing 2% glutaraldehyde for 48 h at room temperature and dehydrated in a graded series of aqueous ethanol solutions (30, 50, 70, 80, 90, 95 and 100% ethanol) for 15 min each. The sections were dried overnight at room temperature, mounted on aluminum stubs and sputter-coated with a gold-palladium alloy under a vacuum for 2 min. At last, the sections were observed using a transmission electron microscopy (Hitachi H-7500).

### Statistical analysis

All data provided in this study were from at least three independent replicates. Significant differences between treatments and controls were determined with an analysis of variance using SAS 8.1 software. The means were compared using Tukey’s HSD test.

## Results

### BcGs1-induced necrosis activity and resistance to *Botrytis cinerea* in tomato

**Fig. 1:**
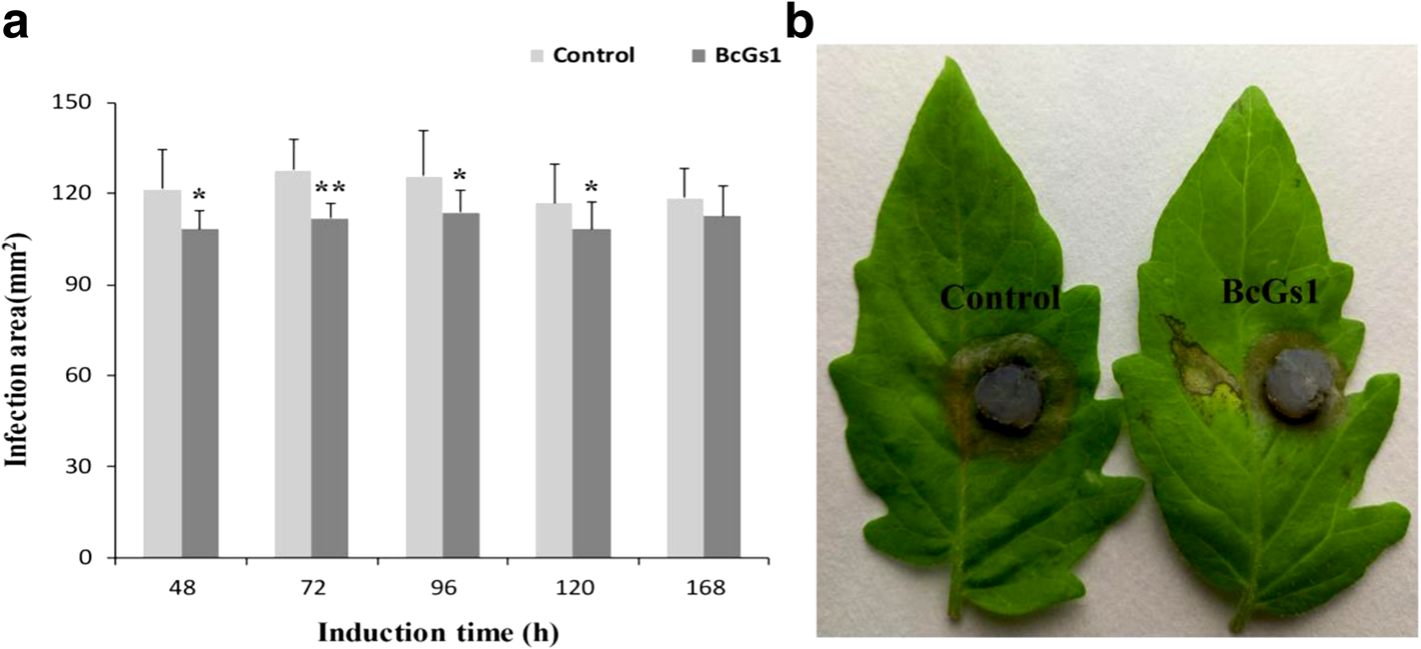
BcGs1-induced disease resistance against *Botrytis cinerea* on detached tomato leaves. **a** Infection area of *Botrytis cinerea* at different induction times post BcGs1 infiltration. Error bars represent the means ±SD in three biological replicates. The asterisks* and ** indicate significance of *p* = 0.05 and *p* = 0.01, respectively. **b** Comparison of *Botrytis cinerea* spot diameter at 72 h between BcGs1 infiltration and control

To investigate the necrosis activity in various host plants, we first obtained protein BcGs1 from the fermentation of *Botrytis cinerea* according to the method of Zhang, et al. [23]. Purified BcGs1, with 72 kDa of relative apparent molecular weight, displayed a single band in the SDS-PAGE (Additional file 1: Figure S1A). The BcGs1 could induce necrosis activity in the tomato, tobacco, cucumber and pea leaves 12 h post BcGs1 infiltration (Additional file 1: Figure S1B), indicating that BcGs1 has rapid necrosis activity in host plants. To analyze the appropriate induction time for disease resistance against *Botrytis cinerea* in tomato, fully mature 4-week-old tomato leaves were infiltrated with 0.25 μM BcGs1 solution at opposite sides of the central vein, and Tris-HCl buffer was used as control. *Botrytis cinerea* disc were inoculated on the detached leaves at 48, 72, 96, 120 and 168 h after BcGs1 induction. The lesion areas at different induction times were measured using the cross method. BcGs1-treated tomato leaves showed a significant reduction in the lesion area caused by *Botrytis cinerea* compared to the Tris-HCl buffer control. The smallest lesion area appeared at 72 h, and the lesion area was reduced 23.3% compared to the control. The result showed that 72 h was the most suitable induction time of BcGs1 (Fig. 1a, b). Therefore, our research showed that BcGs1 induced significant resistance to *Botrytis cinerea* in tomatoes with obvious necrotic activity.

### Two domains of Glyco-hydro 15 (GH15) and CBM20_glucoamylase (CBM20) are required for BcGs1 full necrosis activity

**Fig. 2:**
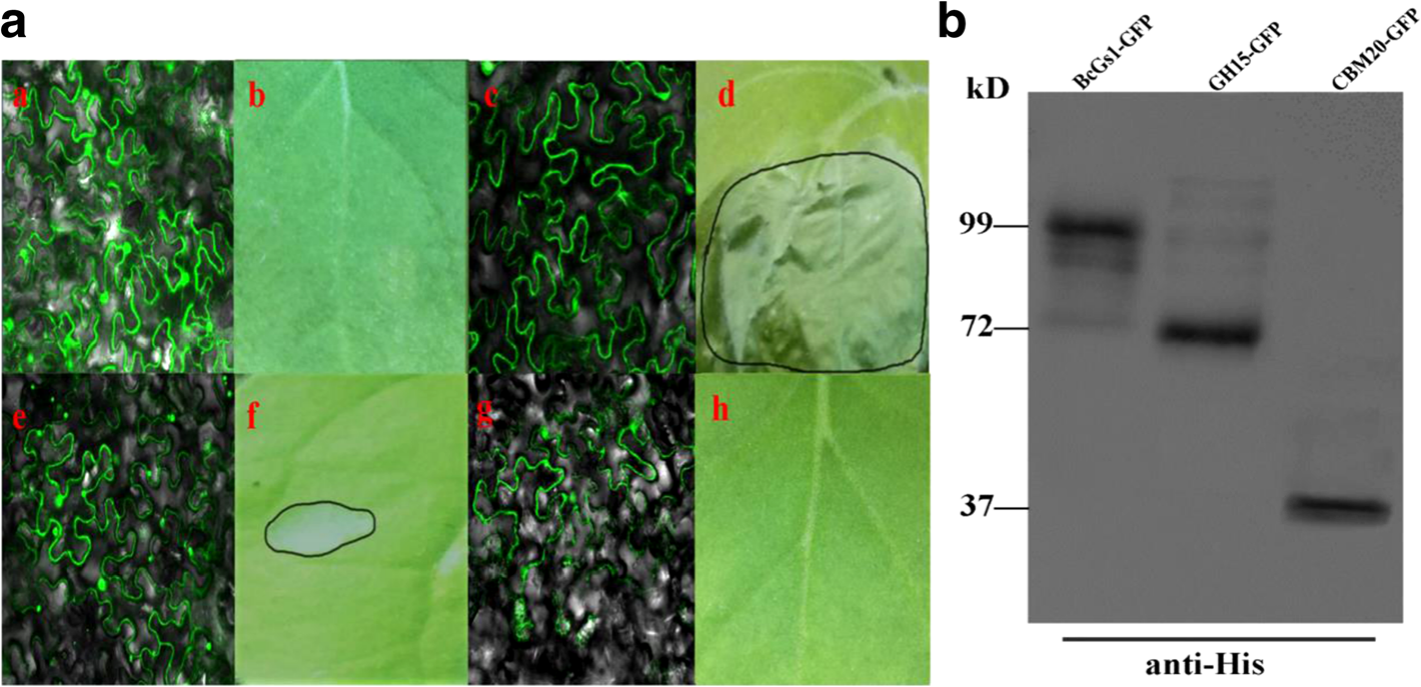
Transient expression and Western Blot detection of BcGs1, GH15 and CBM20. The pYBA1152 vector was used to transiently express these proteins via *Agrobacterium*-mediated inoculation for 48 h in *Nicotiana benthamiana*. **a** a, c, e and g represent the transient expression of the pYBA1152 empty vector, BcGs1, GH15 and CBM20; b, d, f and h represent the necrotic response induced by pYBA1152 empty vector, BcGs1, GH15 and CBM20 protein, respectively. **b** Western Blot verification of the expression of protein BcGs1-GFP, GH15-GFP and CBM20-GFP by His-tag

BcGs1 contains a Glyco-hydro 15 (GH15) domain and a CBM20_glucoamylase (CBM20) domain. To identify the functional domain for necrosis activity, BcGs1, truncated GH15 and CBM20 were expressed transiently in *Nicotiana benthamiana* leaves via an Agrobacterium-mediated transient expression system. Obvious fluorescence under a confocal fluorescence microscope and western blot indicated that BcGs1, truncated GH15 and CBM20 were expressed in *Nicotiana benthamiana* leaves. Transient expression of BcGs1 could induce strong necrosis, whereas GH15 induced a faint necrotic response, and CBM20 had no necrotic response (Fig. 2). The result indicated that the two domains are required for the full necrotic activity of BcGs1.

### Ifferentially expressed protein analysis of iTRAQ

**Fig. 3:**
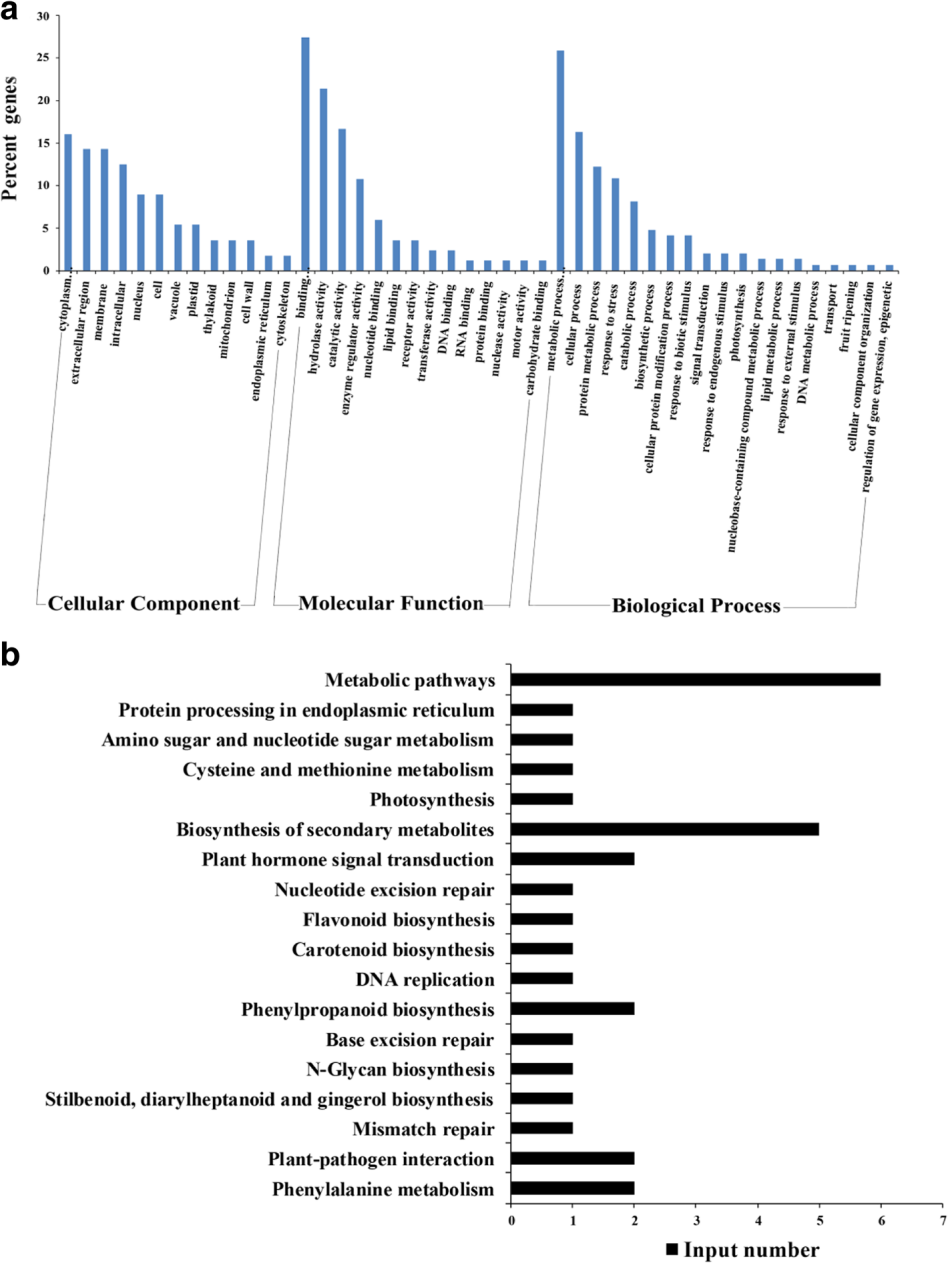
Analysis of the GO function enrichment and KEGG pathway of the differential proteins. **a** Percent of differentially expressed proteins in the cellular components, molecular functions and biological processes; **b** The KEGG pathway enrichment analysis of differential proteins

To investigate the mechanism of BcGs1-triggered defense response in tomato plants, differential proteomics was performed using the iTRAQ technique. A total of 109 proteins were differentially accumulated. Among them, 71 up-regulated proteins with a fold-change > 1.3(*P* < 0.05) and 38 down-regulated proteins with a fold-change < 0.77(*P* < 0.05) were identified, while there were 66 function-known proteins (Table 2). GO-based classification was conducted by Protein accession subjected to InterPro and GO annotation. GO functional analysis showed that enriched proteins were involved in molecular functions of binding, hydrolase activity and catalytic activity, in biological processes of metabolism, cellular protein modification processes, transport, regulation of gene expression, response to stress and stimulus, biosynthetic processes, catabolic processes, signal transduction, and so on (Fig. 3a). KEGG pathway analysis showed these differential proteins enriched in metabolic pathways, biosynthesis of secondary metabolites, phenylpropanoid biosynthesis, phenylalanine metabolism, plant-pathogen interactions and plant hormone signal transduction (Fig. 3b).

**Table 2:** Differential function-known proteins induced by BcGs1 in tomato leaves

UniprotAccession	Protein Name	Fold- change	*P*-Value
B2LW68	PR1 protein	1.566572077	0.000944
K4CWC4	PR10 protein	1.462864947	0.014634
K4CWC5	PR10 protein	1.566549379	0.022764
K4C2B6	Pathogenesis-related ST-2-like protein	3.414710726	0.002007
K4CWC6	Pathogenesis-related STH-2 protein	1.566676247	0.010109
Q9M3X2	Pathogenesis-related protein (PR-5 protein)	1.339407849	0.022775
P32045	Pathogenesis-related protein P2	1.45889008	0.001751
P12670	Protein NP24	1.79280756	0.001972
K4B0B4	wound-induced protein WIN1-like	1.569930049	0.001972
K4D1H1	Basic endochitinase B	1.754832463	0.015711
K4D1H0	Basic endochitinase B	1.449194796	0.010864
K4BTI7	Endochitinase EP3	1.538266581	0.001992
Q7Y0S1	Chitinase	1.783958197	0.025998
Q43778	Glucan endo-1,3-beta-D-glucosidase	1.542061966	0.016393
K4CCI7	Glucan endo-1,3-beta-glucosidase 5	1.481474976	0.006155
Q01413	Glucan endo-1,3-beta-glucosidase B	1.575625293	0.032706
Q4A3Y6	Peroxidase cevi16	1.346195291	0.00248
K4BD54	Peroxidase 51	1.482574377	0.009845
K4BE93	Peroxidase-2 like	1.353913743	0.022862
K4BTH6	Peroxidase 12	1.313139311	0.049815
K4C1Q9	Peroxidase P7	1.49643282	0.005034
K4CQE1	Peroxidase 21	1.456256379	0.009186
K4D1W6	Peroxidase1	1.545711881	0.005047
K4D6T3	Peroxidase	1.589423535	0.002984
K4CG47	Proteasome subunit alpha type	1.3264202	0.02516
K4CFM0	Serine/threonine-protein kinase	1.443769349	0.015025
K4ASR1	Syntaxin-121	1.311591286	0.035885
D6C447	Xanthoxin dehydrogenase	1.751194692	0.000643
Q8RXB6	N-hydroxycinnamoyl-CoA: tyramine N-hydroxycinnamoyl transferase THT7–8	1.406408234	0.017242
K4BCJ8	Patatin	1.412470948	0.015274
K4BV09	Patatin	2.044321442	0.000288
H1ZXA9	Heat shock protein 70 isoform 3	1.348939566	0.036986
E1AZA3	Late embryogenesis abundant protein	1.871127432	0.027053
K4 DC90	Leucine aminopeptidase 1	1.753120542	0.032578
K4DHT1	Dihydrolipoyl dehydrogenase	1.42489934	0.001212
K4CYL4	Cysteine synthase	1.348603246	0.001564
K4BKV2	Enolase 1, chloroplastic	1.35345901	0.028962
K4BDC1	Caffeoyl-CoA O-methyltransferase 1	1.876094119	2.25E-05
K4B172	Calreticulin-2	1.323221668	0.007139
P05116	1-aminocyclopropane-1-carboxylate −2-oxidase 1	1.54366749	0.005854
K4B7W7	40S ribosomal protein S25–2	1.332518921	0.040021
K4BLT8	4-coumarate--CoA ligase 2	1.453093916	0.022281
K4D3M1	60S ribosomal protein L4–1	1.355836521	0.033742
K4BPX5	60S ribosomal protein L6–3	1.321870467	0.031334
K4BXJ9	6-phosphogluconate dehydrogenase, decarboxylating 3	1.377879577	0.022689
K4C740	Alanine aminotransferase 1, mitochondrial	1.406412755	0.011761
K4D7Q7	12-oxophytodienoate reductase 1	0.654045863	0.001084
K4BWB5	30S ribosomal protein S13, chloroplastic	0.76212412	0.000918
Q2MI78	30S ribosomal protein S18, chloroplastic	0.735498477	0.003657
Q2QJT5	ASR4	0.487069561	0.006403
Q0PY39	Auxin repressed/dormancy associated protein	0.573787795	0.000427
K4CV63	Cytochrome b-c1 complex subunit 6	0.699897597	0.029526
Q5NE20	Carbonic anhydrase	0.693825962	0.049301
K4D9P5	DNA ligase	0.661355604	0.022205
G8D593	Ribulose bisphosphate carboxylase large chain (Fragment)	0.610547859	0.030248
P27065	Ribulose bisphosphate carboxylase large chain	0.695991889	0.042018
P08706	Ribulose bisphosphate carboxylase small chain 1, chloroplastic	0.72863624	0.025037
P05349	Ribulose bisphosphate carboxylase small chain 3B, chloroplastic	0.701040127	0.005291
Q3C2L6	Sorbitol related enzyme	0.703582864	0.005221
K4CIE2	Peptidyl-prolyl cis-trans isomerase	0.76180139	0.019223
K4CAM3	Photosystem I reaction center subunit IV B, chloroplastic	0.766668988	0.008467
K4CJ02	Photosystem I reaction center subunit N, chloroplastic	0.664262723	0.047516
Q40163	Photosystem II 10 kDa polypeptide, chloroplastic	0.684317387	0.028599
V5YNW6	Plasma membrane intrinsic protein 21	0.672034247	0.003512
K4BIT3	Polyadenylate-binding protein 1	0.732236793	0.005001
K4D305	Glycine-rich RNA-binding protein 3, mitochondrial	0.768783343	0.002869

### QRT-PCR analysis of the expression profile of genes encoding differential-display proteins

**Fig. 4:**
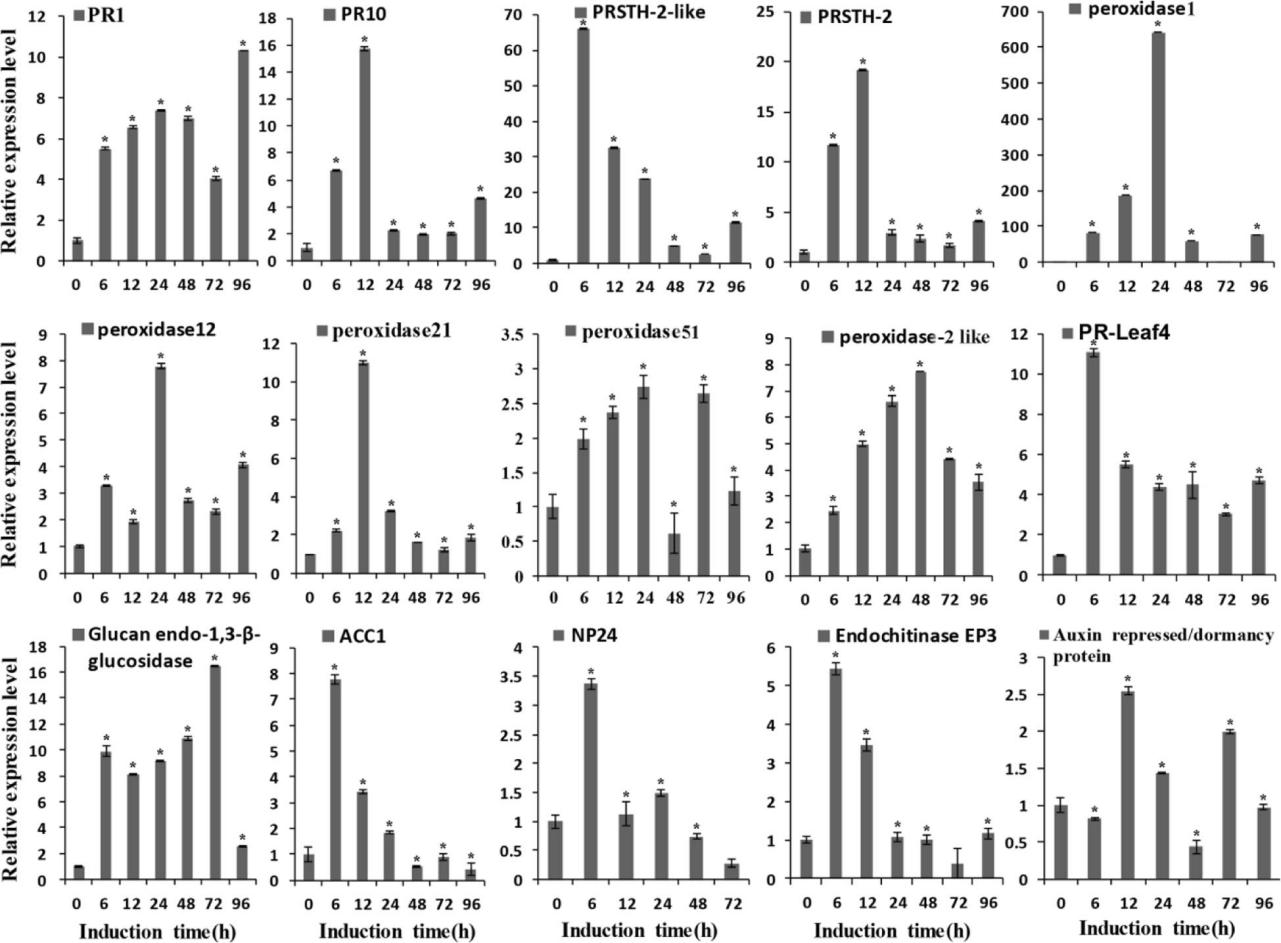
Relative expression level of genes encoding differentially expressed proteins in tomato leaves at various times after BcGs1 induction. Relative expression (±SEM) is the fold-change at the given hours post induction compared with the control and was analyzed using quantitative RT-PCR. Expression levels were normalized to those of actin as a housekeeping gene. Error bars indicate ±SEM. Essentially identical results were obtained for each gene in three independent experiments. The asterisks* indicate significance of *p* = 0.01

Based on the BcGs1-induced differential proteomics analysis, pathogenesis-related proteins, peroxidases, chitins, phenylpropanoid biosynthesis-related proteins, and biosynthesis of secondary metabolite-related proteins were selected and quantitatively confirmed in a biologically independent experiment using qPCR. The relative expression levels of the fifteen genes are shown in Fig. 4. The genes PR1 and PR10 were up-regulated 5–8-fold at 6–12 h. The PRSTH-2-like protein was up-regulated 60~ 70-fold at 6 h, and PRSTH-2 was up-regulated ~ 20-fold at 12 h. The peroxidases we tested had an up-regulation 4–10-fold between 6 and 48 h, especially peroxidase1 (K4D1W6), which was up-regulated 600-fold at 24 h. The 1, 3-β-glucanases and chitinases were up-regulated ~ 10 and ~ 5-fold at 6 h, respectively. The auxin repressed gene was up-regulated 2.5-fold. The antifungal protein NP24 was up-regulated 3.5-fold at 6 h. Ethylene synthesis-related protein ACC1 was up-regulated 8-fold at 6 h. These results suggested that most genes encoding defense response proteins were up-regulated, which was consistent with the proteomics data. Up-regulation of these proteins and genes indicated that BcGs1 activated the basal defense response in tomato and induced the phenylpropane metabolic pathway.

### BcGs1 activates the phenylpropanoid metabolite pathway

**Fig. 5:**
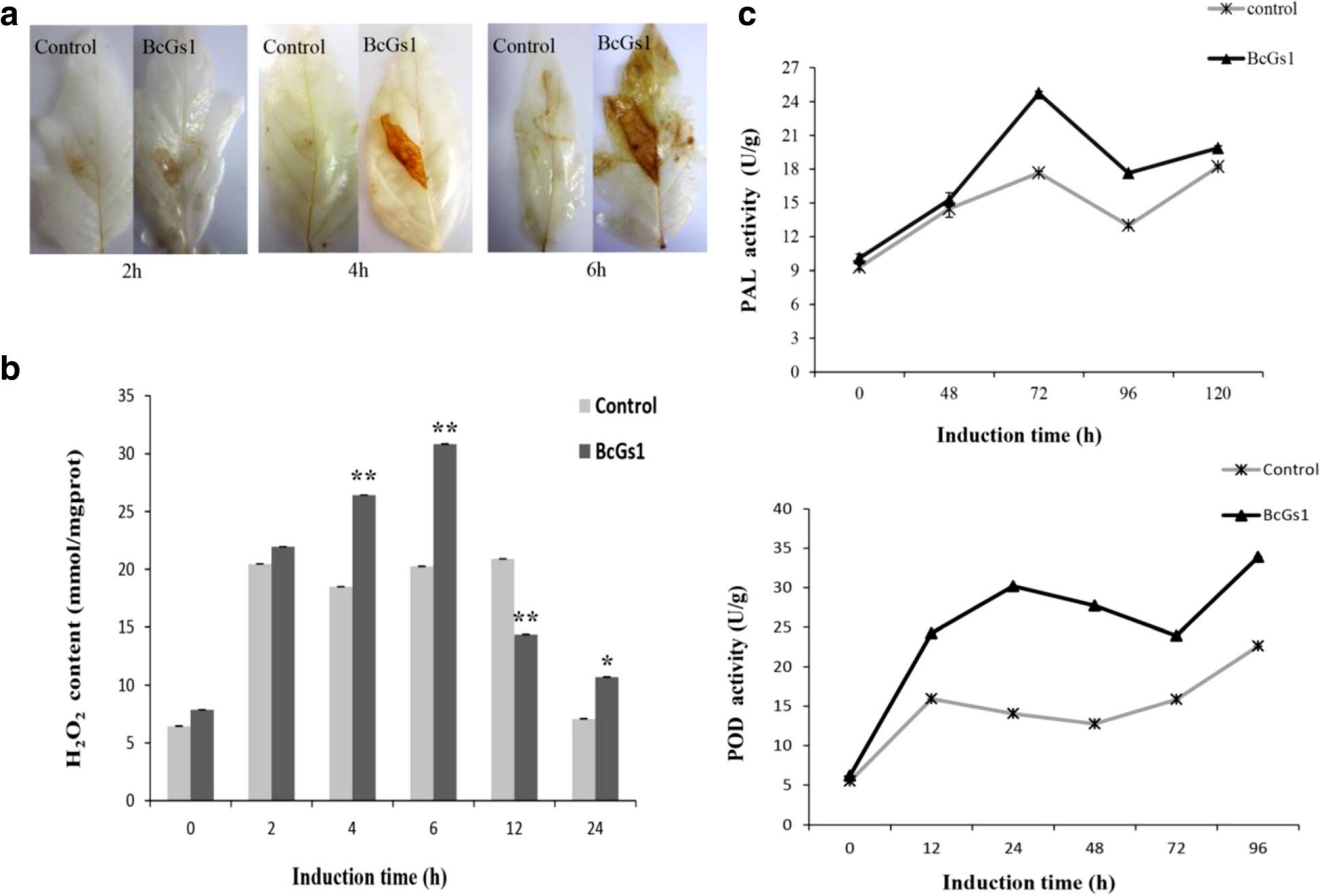
H_2_O_2_ accumulation and the activity of PAL and POD in BcGs1-induced tomato leaves. **a** H_2_O_2_ accumulation at 2 h, 4 h, 6 h after BcGs1 induction; **b** Quantitative determination of H_2_O_2_ content. BcGs1-infiltrated areas were stained brown compared with the buffer control. The asterisks* and ** indicate significance of p = 0.05 and p = 0.01, respectively. **c** Kinetics of PAL and POD activity after BcGs1 treatment of the leaves. The activities were measured 0–120 h after BcGs1 treatment. The values are the mean ± SD of triplicate samples

The ROS production followed pathogen attacks as an essential component in the signal transduction cascade and mediate plant defense response [26]. We detected H_2_O_2_ accumulation at the inoculation site using DAB staining. Brown precipitates were formed in the tomato leaves at 2, 4 and 6 h post BcGs1 infiltration (Fig. 5a). The quantitative analysis showed the H_2_O_2_ accumulation in BcGs1-induced tomato leaves was significantly higher than that of the control at 2, 4, 6, 12 and 24 h post treatment and reached a maximum at 6 h with a 1.5-fold increase (Fig. 5b). According to this data, we suggested that BcGs1 enhanced tomato intracellular production of H_2_O_2_, increased the extracellular peroxidase activities, and further generate monolignol phenoxy radicals that couple spontaneously to form lignin polymers.

The phenylpropanoid metabolite pathway is an important indicator of plant basal defense. The activities of the enzymes PAL and POD are involved in the phenylpropanoid pathway leading to the synthesis of lignin. Accordingly, PAL and POD were measured after BcGs1 infiltration (Fig. 5c). The results showed that activities of PAL and POD increased 1.5 and 2-fold compared to the untreated leaves, respectively. The data indicated that the phenylpropanoid pathway might be activated and play a defensive function in tomato.

### BcGs1 enhances secondary synthesis of lignin and reinforces the cell wall

**Fig. 6:**
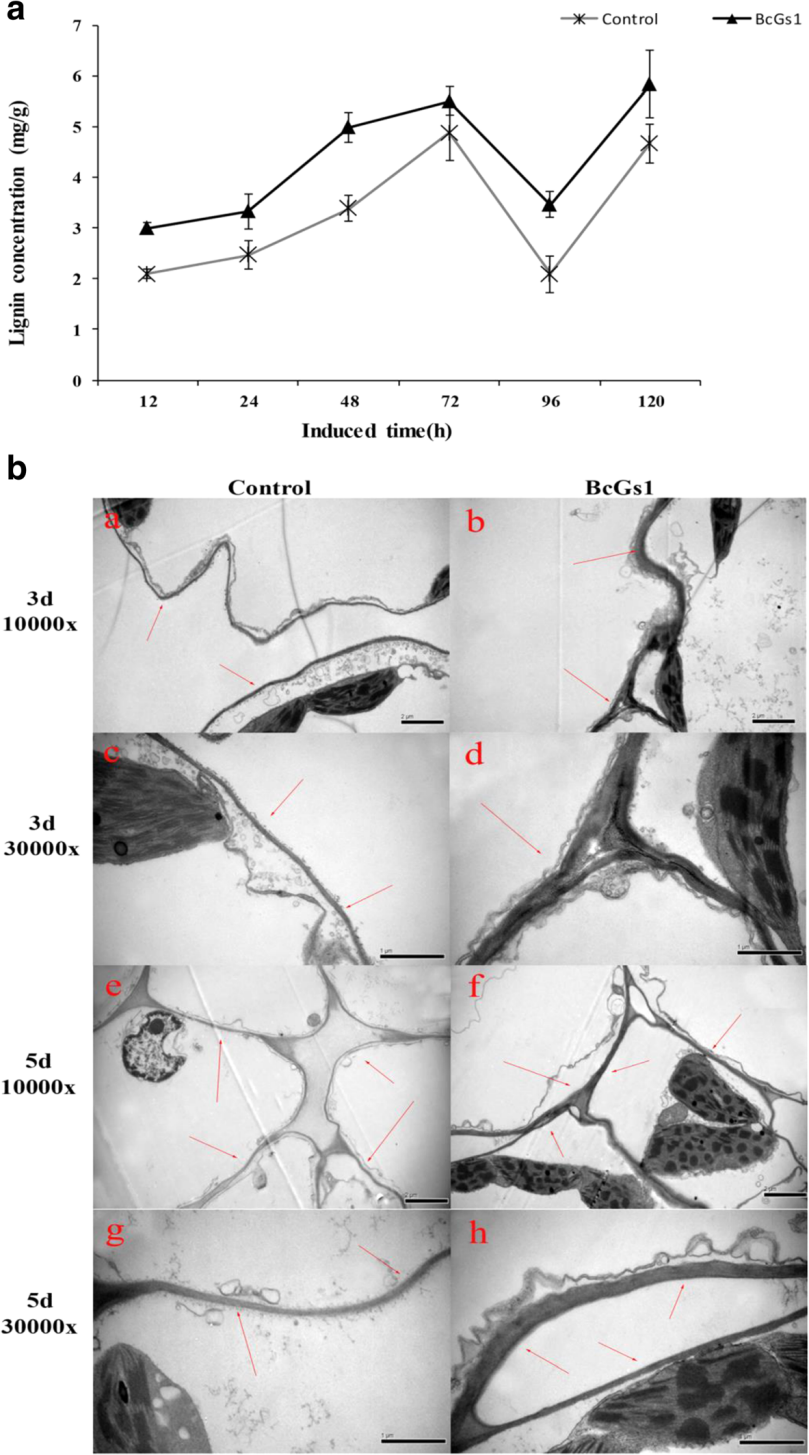
Lignin accumulation and cell wall thickening induced by BcGs1 in tomato leaves. **a** Lignin accumulation was measured at 12, 24, 48, 72, 96 and 120 h, and the data are the mean ± SD from three independent experiments. **b** Electron microscopy observation of the cell wall in the BcGs1-treated tomato leaf and control. a, b, e, and f were observed at 10000×; c, d g, and h were observed at 30000×. a and c, e and g were the control at 3 days and 5 days, respectively; b and d, f and h were treated by BcGs1 at 3d and 5d, respectively. Red arrows point to the cell wall

Lignin played a critical role in the plant response to pathogen infection. The synthesis and deposition of lignins were assumed to be physical barriers that made the cell walls more resistant to mechanical pressure during fungal penetration [25, 27]. BcGs1 elicitor-treated plants exhibited 1.5-fold increases in lignin deposition compared to the control at 48 h after elicitor treatment (Fig. 6a).

Cell wall strengthening played an important role in plant disease resistance [25]. Our results showed that the cell wall in the BcGs1-treated leaves was obviously thickened compared with the Tris-HCl control at 3 days and 5 days (Fig. 6b). High levels of lignin and cell wall thickness could enhance the toughness and mechanical strength of the cell wall, protect the differentiated cells, and reduce cells gap, leading to resistance to pathogen infection [22, 27].

## Discussion

Successful pathogen infection must break physical barriers and chemical defenses in plants. Plant cell walls are an important barrier against pathogen attack regardless of the biotrophic and necrotrophic pathogens. To overcome plant defense, the pathogen secretes various types of CWDEs to degrade the plant cell wall. CWDEs are conserved in extensive pathogens and recognized as PAMPs or DAMPs to initiate PTI. BcGs1, a CWDE secreted from *Botrytis cinerea*, could induce the defense response and improve disease resistance to *Botrytis cinerea* in tomato [23]. However, the mechanism is unclear. To investigate the difference in the defense response of BcGs1 treated plants and control plants, proteomics was applied to analyze the differential proteins. Forty-six up-regulated and 20 down-regulated proteins were detected. PR proteins, chitinases, peroxidases, 1, 3-β-glucanases and secondary metabolic related proteins were enriched based on Go analysis and KEGG analysis.

Pathogenesis-related (PR) proteins have been used as biomarkers of plant defense responses [28] and have been distinguished entities for system acquired resistance (SAR) in plants [29]. Based on the PRs functional properties and structure, 17 different PR protein families have been characterized, and these PR families have a wide range of functions from making the cell wall more rigid to signal transduction and antimicrobial activity [30]. In our present study, 16 PR proteins, including PR1, PR5, PRP2, PR10, PRSTH-2/PRSTH-2-like proteins, chitinases and glucan 1, 3-β-glucosidases, were significantly induced in BcGs1-treated tomato leaves. PR1 proteins have antifungal activity in tomatoes and tobacco, but the mechanism remain unknown [31]. PR5 proteins include thaumatin, osmotin and related proteins, many of which also have antimicrobial activity [32]. The PRP2 protein, a member of the proline-rich protein family, was a structural cell wall protein and accumulated in response to fungal pathogen attack and *Phytophthora megasperma f. sp. glycinea* elicitors [33, 34]. H_2_O_2_ mediated the oxidative cross-linking of the PRP2 protein into the wall structure. Moreover, the PRP2 protein also regulated cell wall properties and was involved in development and defense [35]. PRSTH-2, belonging to the PR-10 family, was regulated by fungal elicitors, plant hormones and defense-related signaling molecules [30, 36]. Chitinases were released in the early stage of pathogen infection and could hydrolyze fungal cell wall chitin, when hyphae penetrated the intercellular space, to inhibit its growth [37]. Glucan 1, 3-β-glucosidases belong to PR-2 protein family, with a function to hydrolyze β-1, 3-glucans, which are major structural compounds of fungal cell walls [38]. Increased expression of PR protein information indicated that BcGs1 induced the tomato basal defense, including cell wall reinforcement, biosynthesis of antimicrobial phytoalexin and phytohormone.

Peroxidase was divided into two types: class I and class III; class I is intracellular, while class III is secreted into the cell wall or the surrounding medium [39]. Class III comprises the secretory plant peroxidases, which have multiple tissue-specific functions, such as the removal of hydrogen peroxide from chloroplasts and cytosol, biosynthesis of the cell wall, and wounding defense responses [40]. In our experiment, eight differential expression peroxidases were detected, including peroxidase (K4D6T3), peroxidase1, peroxidase12, peroxidase21, peroxidase cevi16, peroxidase-2 like, peroxidaseP7, and peroxidase51, and they all belong to class III. Peroxidases can prevent excessive accumulation of H_2_O_2_, remove H_2_O_2_ and mediate many H_2_O_2_-related defense responses [26]. Peroxidases are also involved in phenolic metabolism and biosynthesis of lignin from cinnamyl alcohols and other polymers [41–43]. Overexpression of a basic peroxidase in transgenic tomato plants exhibited a higher lignin content than the wild-type plants [44, 45]. Individual *Arabidopsis* peroxidase (*AtPrx*) gene families are also involved in both cell growth and lignification in a variety of tissue types, including stems, leaves, and roots [46–49]. The defensive action of peroxidases is probably accompanied by the activation of other defense enzymes, such as β-1, 3-glucanase and chitinases [39]. Although the function of peroxidase is ambiguous, the role of peroxidases in the detoxification of ROS and lignification, as well as the efficient coupling of the two processes, was acknowledged [44]. Therefore, our results demonstrated that the accumulation of peroxidases decreased the cell-wall plasticity by the lignification of the cell in the response to BcGs1 stress.

ROS production was one of the earliest defense responses against pathogen invasion in plant-pathogen interactions [50, 51]. H_2_O_2_ accumulation at the site of pathogen infection has been shown to be decisive for the outcome of tomato-pathogen interactions [52]. It could cause direct pathogen destruction, trigger hypersensitive cell death, activate defense response-related genes, or serve as a secondary messenger in the systemic signaling network of the plant cell [50, 53–55]. Furthermore, H_2_O_2_ was also found to be critical for determining the resistance of tomato to *Cladosporium fulvum*, anthracnose fungus and powdery mildew fungus [56–58]. H_2_O_2_ accumulation in the epidermal cell layer was accompanied by an increase in the extracellular peroxidase activities, and the peroxidases mediated many H_2_O_2_-related defense responses and caused cell wall modification [26]. A severe accumulation of H_2_O_2_ was observed in BcGs1-treated tomato leaves at 24 h and increased 1.5-fold at 6 h. Moreover, lignin accumulated after 6 h of treatment by BcGs1. These results showed that BcGs1 activated H_2_O_2_-related defense responses, resulting in lignin accumulation.

### Biosynthesis of secondary metabolites related proteins

Secondary metabolites play a fundamental role in the plant’s fight against pathogen infection. Based on the biosynthesis substrates and pathways, phenylpropanoid, nitrogen-containing substances and terpenoid pathways were classified [59]. Terpenoid and quinone compounds could enhance plant disease resistance. Multiple branches of the phenylpropanoid pathway have been reported for various model plants, including tomato, rice, *Arabidopsis* and legume plants [60–62]. Many phenylpropanoids exhibit broad-spectrum antimicrobial activity and help the plant fight microbial disease. In our study, the key protein 4-coumarate-CoA ligase 2 (4CL) and Caffeoyl-CoA O-methyltransferase 1 (CCoAMT1) synthesize the G-lignin monomer in the branch where the phenylpropanoid pathway was identified. Previous results found that CCoAMT down regulation in *alfalfa* resulted in the reduction of G lignin units [63]. The two essential proteins and encoding genes were significantly up-regulated post-BcGs1 infiltration, indicating that BcGs1 activates the phenylpropanoid pathway in the tomato.

### Lignin formation and cell wall histochemical localization

Lignin, a major component of the secondary cell wall of plants, is an important part of the defense against the penetration of invading pathogens [64–66]. Lignin can enhance the mechanical strength, alter the compressibility and porosity of the cell wall and form a barrier against the infection of pathogens [64, 67–69]. In the present study, differences in lignin content between BcGs1-treatment and Tris-HCl buffer-treated leaves were analyzed, and the results showed that the lignin content in BcGs1-treated leaves exhibited a 1.5-fold increase compared to the control, indicating that it might restrict *Botrytis cinerea* spread in tomato. Moreover, the key enzymes involved in this pathway, such as PAL, POD, 4CL and CCoAMT1, were detected after BcGs1 was induced in tomato leaves. These observations were confirmed via the transcriptionally up-regulated expression of the PAL, POD 4CL and CCoAMT1 genes compared to the control. Furthermore, histochemical localization showed that the cell wall of BcGs1-treated leaves was significantly thickened post BcGs1 treatment compared to the control. Cell wall thickening enhanced the mechanical strength and improved disease resistance to *Botrytis cinerea* infection.

## Conclusions

BcGs1 could significantly activate disease resistance against *Botrytis cinerea* at 72 h post-induction. Two domains are required for BcGs1 full necrosis activity. Differential expression proteins were identified in tomato plants using a proteomics approach. PR proteins, peroxidases, Glucan endo-1, 3-β-glucosidase, chitinases, ethylene synthesis-related proteins, and biosynthesis of secondary metabolites were involved in BcGs1-induced resistance. BcGs1-infiltrated tomato plants exhibited H_2_O_2_ accumulation, increased levels of regulation of the key PAL and POD enzymes, lignin end-product accumulation in the phenylpropanoid pathway, and cell wall thickening. According to BcGs1-induced PR differential expression proteins, peroxidase and the biosynthesis of secondary metabolites, the mechanisms by which BcGs1 triggers disease resistance against *Botrytis cinerea* were summarized as follows. BcGs1 induced H_2_O_2_ production, which not only increased peroxidase activity but also caused cell wall strengthening and lignin accumulation. Meanwhile, many defense-related proteins were up-regulated, including PRs, Glucan endo-1, 3-β-glucosidase and chitinase. By combining biochemical and histochemical data, we suggested that BcGs1 triggers up-regulation of the phenylpropanoid pathway-related genes and protein, enzyme activities, high lignin levels and cell wall thickness, and ultimately generates tomato disease resistance. Overall, lignin metabolism played a critical role and was involved in BcGs1-induced defense response in the tomato.

## Additional file

Additional file 1: **Figure S1.** Purification and necrosis activity of the protein BcGs1. A: SDS-PAGE analysis of purified BcGs1. M, Protein marker. 1, Purified BcGs1. B: Necrosis activity of BcGs1 in tomato, tobacco, cucumber and pea leaves. (DOCX 673 kb)

## Abbreviations

4CL: 4-coumarate-CoA ligase 2
CCoAMT1: Caffeoyl-CoA O-methyltransferase 1
CWDE: cell wall-degrading enzyme
DAB: nitro 3, 3′-diaminobenzidine
PAL: phenylalanine-ammonia lyase
PAMP: pathogen-associated molecular patterns
POD: peroxidase
PR: pathogenesis-related proteins
PTI: microbe-associated molecular pattern (MAMP)-triggered immunity
ROS: reactive oxygen species
TGA: thioglycolic acid
