# Supplementary figures and images for: Lignin metabolism involves Botrytis cinerea BcGs1- induced defense response in tomato
Source: BMC Plant Biol. 2018 Jun 4;18:103. doi: 10.1186/s12870-018-1319-0 (PMC5987389; doi:10.1186/s12870-018-1319-0)

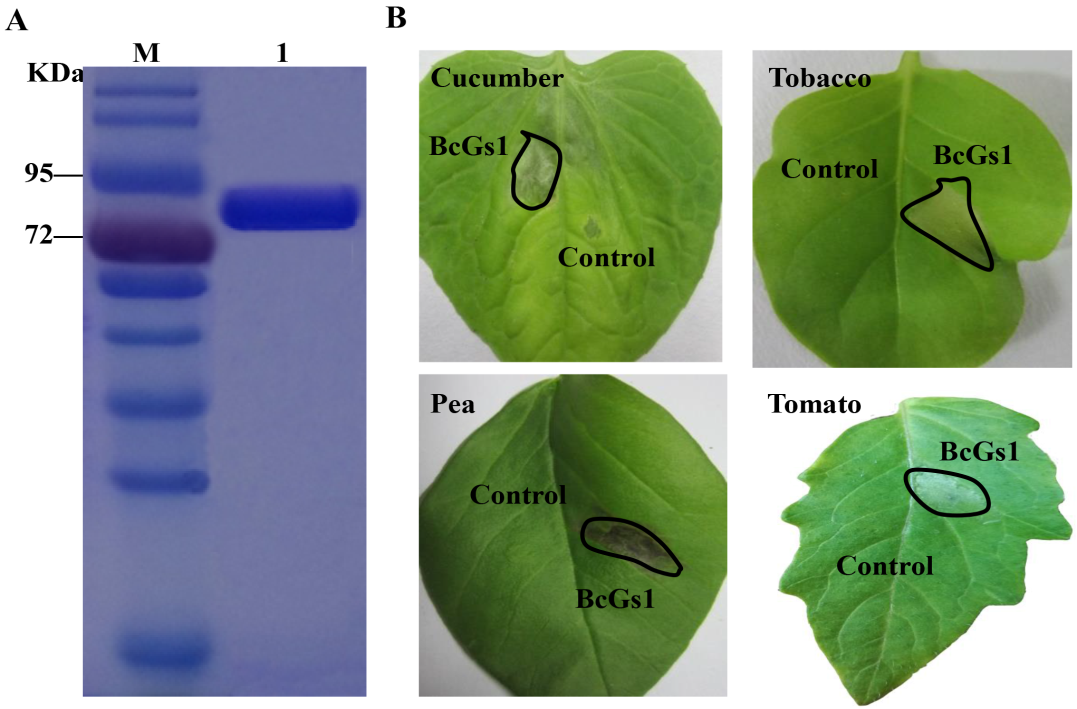

Supplement: Supplementary file 1 — Figure S1. Purification and necrosis activity of the protein BcGs1. A: SDS-PAGE analysis of purified BcGs1. M, Protein marker. 1, Purified BcGs1. B: Necrosis activity of BcGs1 in tomato, tobacco, cucumber and pea leaves. (DOCX 673 kb) [file 12870_2018_1319_MOESM1_ESM.docx]
